# Macrophage Receptor With Collagenous Structure Polymorphism and Recurrent Respiratory Infections and Wheezing During Infancy: A 5-Years Follow-Up Study

**DOI:** 10.3389/fped.2021.666423

**Published:** 2021-07-27

**Authors:** Francesco Savino, Francesco Pellegrino, Valentina Daprà, Cristina Calvi, Carla Alliaudi, Paola Montanari, Ilaria Galliano, Massimiliano Bergallo

**Affiliations:** ^1^Early Infancy Special Care Unit, Regina Margherita Children Hospital, Azienda Ospedaliera Universitaria Città della Salute e della Scienza di Torino, Turin, Italy; ^2^Post Graduate School of Pediatrics, University of Turin, Turin, Italy; ^3^Department of Public Health and Pediatric Sciences, Paediatric Laboratory, Medical School, University of Turin, Turin, Italy

**Keywords:** atopy, MARCO polymorphisms, SNPs, rs1318645, recurrent wheezing

## Abstract

**Background:** Recurrent wheezing is a common clinical manifestation in childhood, and respiratory syncytial virus infection is a well-known risk factor. However, the genetic background favoring the development of recurrent wheezing is not fully understood. A possible role of macrophage receptor with collagenous gene (MARCO) polymorphism has been recently proposed.

**Objective:** To investigate a correlation between MARCO rs1318645 polymorphisms and susceptibility to recurrent wheezing during childhood.

**Methods:** We prospectively recruited 116 infants, of which 58 with respiratory syncytial virus bronchiolitis and 58 controls hospitalized at Regina Margherita Children's Hospital, Turin, Italy, between November 2014 and April 2015. All subjects were investigated for MARCO rs1318645 polymorphisms in the first period of life. Genotyping of rs1318645 was carried out by TaqMan mismatch amplification mutation assay real-time polymerase chain reaction procedure. Subjects were then enrolled in a 5-year follow-up study to monitor the occurrence of wheezing and respiratory infections.

**Results:** The analysis of MARCO rs1318645 of allelic frequencies shows an increasingly significant risk to develop recurrent infection (*p* = 0.00065) and recurrent wheezing (*p* = 0.000084) with a wild-type C allele compared with a G allele. No correlation was found between wheezing and past respiratory syncytial virus infection (*p* = 0.057) and for a history of atopy in the family (*p* = 0.859).

**Conclusion:** Our finding showed that subjects with C allelic MARCO rs1318645 polymorphism are at higher risk for recurrent infection and wheezing episodes during the first 5 years of life. Future studies of genetic associations should also consider other types of polymorphisms.

## Introduction

Recurrent wheezing and consequently associated asthma are common respiratory disorders during childhood. A rise in prevalence and severity has been observed in recent years, contributing to increased health system costs (1). Historically, respiratory syncytial virus (RSV) has long been considered the most common trigger of recurrent wheezing (2), particularly in preterm infants (3). A recent meta-analysis performed by Liu et al. (4) also suggests an association between early life rhinovirus infections and the subsequent development of wheezing and asthma.

The variation in response to RSV infection suggests that host intrinsic factors influence susceptibility mechanisms and severity of symptoms. Recently, candidate gene single-nucleotide polymorphisms (SNPs) have been associated with RSV disease severity in infants (5).

We recognized macrophage receptor with collagenous structure (MARCO), an innate immunity scavenger receptor, as a possible gene candidate to RSV disease susceptibility. In the MARCO promoter, we identify a polymorphic region, the SNP rs1318645, −156 C/G (C is the wild type), which influences the transcriptional activity of the gene.

MARCO is a class A scavenger receptor, expressed on macrophages membrane, where it recognizes pathogen-associated molecular patterns and environmental or un-opsonized particles. Studies have demonstrated that MARCO and other scavenger receptors decrease the proinflammatory environment in infections, mediating the clearance of lung pathogens (6).

MARCO promoter contains a polymorphic region that, when deleted, reduced transcriptional activity. In enrolled children, we study SNPs rs1318645. The mutation from C (wild type) to G at the −156 sites has been associated with a deletion of an antioxidant response element of the MARCO promoter. That is a binding site for a transcription factor named “nuclear factor erythroid-derived 2-like 2,” also known as NRF2.

TaqMan mismatch amplification mutation assay (TaqMAMA) combines the quantitative strengths of TaqMan with the allele-specific polymerase chain reaction (PCR) of mismatch amplification mutation assay (MAMA). This technique is used for screening human DNA samples for known genetic polymorphisms and may be suitable for broad and cost-effective genotyping applications in all types of laboratories.

Using the MARCO rs1318645 TaqMAMA-specific real-time PCR (7), the outcome of this study is to determine a correlation between MARCO rs1318645 SNP and incidence of bronchiolitis, RSV susceptibility. Consequently, as respiratory infections during infancy, especially by RSV, constitute a significant risk factor for the development of recurrent wheezing and asthma in childhood (8), with a 5-year follow-up, we tried to verify the potential correlation of MARCO rs1318645 SNP and the onset of recurrent infection and wheezing during childhood.

## Materials and Methods

### Population and Enrollment

This case–control study was conducted in Turin Children Hospital Regina Margherita, Italy, between November 2014, April 2015, and December 2020. Study participants were full-term infants with age younger than 12 months. In the control group, we enrolled at term infants, adequate for gestational age (birth weight: 2,500–4,000 g), exclusively or predominant breast milk feeding, who were visited during outpatient control (follow-up visit and routine check) in our Department of Pediatrics at Regina Margherita Children Hospital, Turin, Italy. In the case group, we enrolled infants with a diagnosis of bronchiolitis made by trained pediatricians, hospitalized, and clinically monitored every day.

The parents of the enrolled infants were informed about the purpose, benefits, and possible risks of the study, and written informed consent was obtained. Exclusion criteria included known or suspected impairment of immunological function, major known congenital airway malformations, chronic lung disease, cardiac disease, prematurity (gestational age younger than 37 weeks), neuromuscular disorders affecting swallowing, and known or suspected coagulation disorders or bleeding tendency. Baseline population characteristics (age, sex, breastfeeding history, smoke exposition, family history of atopy, like atopic dermatitis, recurrent wheezing, asthma, and food allergy) reported by parents were collected by investigators. A pediatrician investigator monitored the clinical evolution of the participants by visiting them 5 years after enrollment.

The parents of the enrolled infants were informed about the purpose of the study to obtain written consent, and a standardized questionnaire was administered to investigate the onset of symptoms associated with recurrent wheezing and history of recurrent infections. The pediatrician investigators also asked for information about the development of allergic symptoms, discovering of food allergy, or execution of allergy test (e.g., Patch test or Prick test). The data reported by parents were collected and classified in the previous database.

The study was conducted in accordance with the International Council for Harmonization of Technical Requirements for Registration of Pharmaceuticals for Human Use guidelines protocol, applicable regulations and guidelines governing clinical study conduct, and the ethical principles that have their origin in the Declaration of Helsinki^1^.

### Genetic Marker Selection and Samples Preparation

A MARCO rs1318645 SNP (GenBank access number NT_005403.18)^2^ on chromosome 2 showing a C/G transition in position 24445994 was chosen.

Infants were subjected to a venous blood sample from 7:30 to 8:30 AM during the routine clinical blood sampling to reduce the stress of infants.

Genomic DNA was extracted from blood using the Maxwell16 LEV Blood DNA kit with automatic extractor Maxwell16 System (Promega, Madison, Wisconsin, USA) following the manufacturer's instructions.

Genotyping of rs1318645 was carried out by TaqMAMA real-time PCR procedure (MARCO rs1318645 cod. PP-036) (BioMole, Turin, Italy) (7).

The work described was carried out in accordance with the code of ethics of the World Medical Association (Declaration of Helsinki) for experiments involving humans^1^.

### TaqMan Mismatch Amplification Mutation Assay rs1318645 Real-Time Polymerase Chain Reaction Assays

MAMA allele-specific forward primers were designed. The last nucleotide at 3′ region gives the specificity of the primers. An additional deliberate mismatch in the third last from the 3′ end improves the specificity of MAMA primers. The established assays use the same probe and reverse primer and probe mix PP-036 (BioMole, Turin, Italy). The Probe is a TaqMan labeled at the 5′ end with 6-carboxyfluorescein and at the 3′ with 5-carboxytetramethylrhodamine.

PCR was set up in a volume of 20 μl, containing 5 μl of DNA and 15 μl of reaction mix (PP-036, BioMole, Turin, Italy). Assays were performed with 7500 Real-Time PCR System (Thermo Fisher Scientific, Waltham, Massachusetts, USA), following the standard Life Technologies profiles and subsequently analyzed using ABI Prism 7500 SDS software version 2.0 (Life Technologies, Thermo Fisher Scientific, Waltham, Massachusetts, USA).

### Statistical Analysis

For statistical analysis, the chi-square test was performed using commercial software (GraphPad Prism 5 San Diego, California, USA); a *p* < 0.05 was considered statistically significant. The *P*-value was calculated for each allele in the control group and in patients with recurrent infections and wheezing. A *P*-value of <0.05 was considered significant with 95% confidence intervals.

## Results

### Population

**Figure 1:**
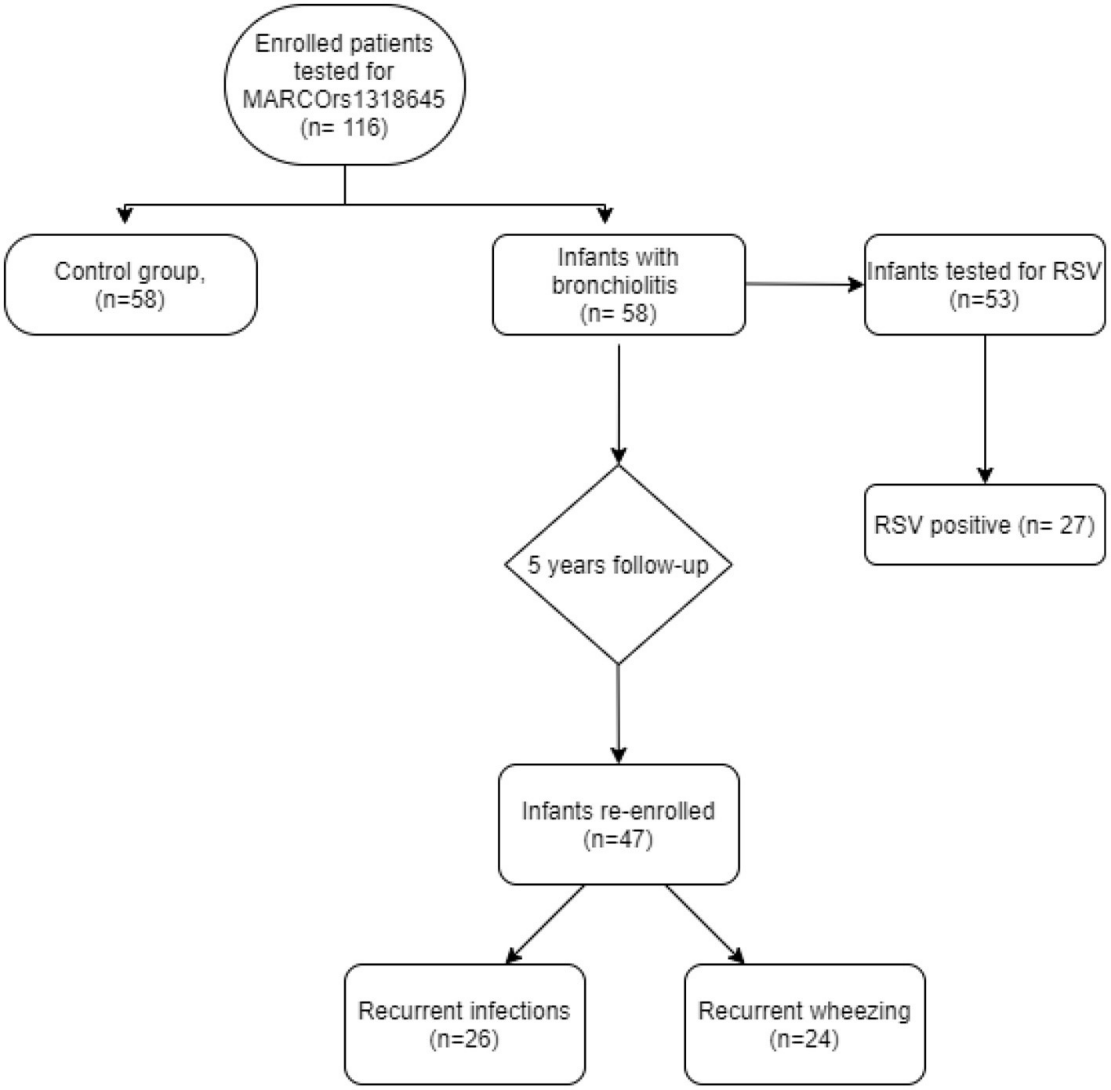
Enrollment process and follow-up.

A total of 116 infants were enrolled. According to the criteria described in section Materials and Methods, 58 infants with bronchiolitis were identified in the case group and tested for MARCO. Bronchiolitis group includes 29 (50%) male patients and 29 (50%) female patients as shown in Figure 1.

Fifty-eight children were enrolled in the control group, including 30 (51.7%) male patients and 28 (48.3%) female patients, at term, adequate for gestational age (birth weight: 2,500–4,000 g), exclusively or predominant breast milk feeding.

As shown in Table 1, both groups were similar (*p* > 0.05 indicates that no statistical effect was observed) for age at enrollment, sex, and type of feeding (breastfeeding). The mean duration of hospitalization in the bronchiolitis group was 6.87 days (range 5–21).

**Table 1:** Epidemiological characteristics of enrolled subjects.

	**Infants with bronchiolitis**	**Healthy subjects**	***P*-value**
	**(*n* = 58)**	**(*n* = 58)**	
Age at enrollment in weeks (mean range)	14.6 (2–24)	13.0 (2–31)	0.830^*^
**Gender**			
Female, *n* (%)	29 (50.0)	28 (48.2)	0.853
Male, *n* (%)	29 (50.0)	30 (51.7)	
Breastfeeding *n* (%)	24 (41.3)	32 (55.2)	0.137
**Nationality**			
Italian, *n* (%)	26 (44.9)	30 (51.7)	0.552
Foreign, *n* (%)	32 (55.1)	28 (48.3)	

* *Mann–Whitney test*.

### Respiratory Syncytial Virus Test Results

In the bronchiolitis group, we executed nasopharyngeal swabs for respiratory viruses. We study RSV susceptibility in 53 (91.4%) infants with bronchiolitis. A total of 27 (51%) tested positive for RSV. Five of 27 were also positive in nasopharyngeal pathogen detection for other viruses (rhinovirus and coronavirus NL63).

Infants with RSV-positive bronchiolitis (51%) and RSV-negative bronchiolitis (49%) have shown the same allelic and genotype frequencies: χ^2^ analysis of genotypes (*p* = 0.373) and allelic (*p* = 0.250); frequencies showed no association between MARCO rs1318645 and RSV susceptibility.

### Five-Year Follow-Up

In bronchiolitis cohort follow-up, we contacted families of bronchiolitis-group infants to organize a visit to our hospital. A team of pediatricians performed a medical examination of enrolled children and interviewed parents using a structured questionnaire.

The follow-up aimed to verify the possible association between MARCO rs1318645 SNP and the onset of recurrent respiratory infections, defined as one or more admissions to the pediatric emergency department for acute respiratory symptoms or three or more visits to general pediatricians for clinical respiratory infections and/or the development of recurrent wheezing [three or more episodes, which requires one or more accesses to the pediatric emergency department or pneumology or allergology specialistic visit and leading to bronchodilator drug (β2 agonist) or inhaled corticosteroids prescription].

Families who referred a history of recurrent wheezing focused on the necessity to access to the emergency department or consult an allergology or pneumology specialist and leading to bronchodilator drug (β2 agonist) or inhaled corticosteroids prescription.

We asked parents about smoke exposition and the development of airway malformations. We excluded two infants who developed airway complex chirurgical conditions. We correctly reenrolled 47 patients, in which we researched family history of atopy (atopic dermatitis, recurrent wheezing, history of asthma, and food allergy in parents).

A total of 19 (40.4%) infants were exposed to passive smoke during infancy. Eleven (57.9%) of these smoke-exposed children developed recurrent airway infections, but also 15 (54%) of 28 nonexposed children developed recurrent respiratory infections. No correlation between cigarette smoke exposition and recurrent infections resulted.

Nine (47.4%) of smoke-exposed children and 15 (54%) of nonexposed children developed recurrent wheezing. No correlation between cigarette smoke exposition and recurrent infections (*p*-value 0.770) or wheezing (*p*-value 0.676) resulted.

Nineteen children (40.4%) referred a history of atopy in their family (e.g., atopic dermatitis, recurrent wheezing, history of asthma, and food allergy). A total of 12 (63.2%) of these presented recurrent infections (*p*-value 0.373) and 10 (52.6%) referred recurrent wheezing (p-value 0.859).

Twenty-four children with past RSV-positive tampons were correctly enrolled in the follow-up. A total of 15 (62.5%) children referred recurrent airway infection, and the same number referred recurrent wheezing. No correlation between RSV infection during infancy and recurrent infections (*p*-value 0.191) or wheezing (*p*-value 0.057) resulted.

### Role of Macrophage Receptor With Collagenous rs1318645 in Recurrent Airway Infections During Childhood

We defined recurrent infections as one or more admissions to the pediatric emergency department for acute respiratory symptoms or three or more visits to general pediatricians for the same symptoms. Allelic and genotype frequencies in an infant with recurrent infections are shown in Table 2. The analysis of allelic frequencies shows a significant risk of C allele to develop recurrent infection compared with G (*p*-value = 0.001).

**Table 2:** MARCO rs1318645 and recurrent airway infections susceptibility.

**Target**	**SNP**	**Alleles/** **genotypes**	**Recurrent infections** ** (*n* = 26)**	**No recurrent infections** ** (*n* = 21)**	***p*-value**
MARCO	rs1318645	C	38 (73.1%)	16 (38.1%)	0.001^*^
		G	14 (26.9%)	26 (61.9%)	
MARCO	rs1318645	CC	13 (50.0%)	5 (23.8%)	0.002^#^
		CG	12 (46.2%)	6 (28.6%)	
		GG	12 (46.2%)	10 (47.6%)	

* *Allelic frequencies;*

# *genotype sequences*.

### Role of Macrophage Receptor With Collagenous rs1318645 in Recurrent Wheezing

We define recurrent wheezing as three or more episodes that require one or more access to the pediatric emergency department or pneumology or allergology specialistic visit and leading to bronchodilator drug (β2 agonist) or inhaled corticosteroids prescription. Allelic and genotype frequencies in an infant with recurrent wheezing are shown in Table 3. The analysis of allelic frequencies shows a significant risk of C allele to develop recurrent wheezing episodes compared with G (*p*-value = 0.001). χ^2^ analysis of genotypes frequencies are significant (*p*-value = 0.002).

**Table 3:** MARCO rs1318645 and recurrent wheezing during childhood.

**Target**	**SNP**	**Alleles/** **genotypes**	**Wheezing** ** (*n* = 24)**	**Not wheezing** ** (*n* = 23)**	***p*-value**
MARCO	rs1318645	C	37 (77.1%)	17 (37.0%)	0.001^*^
		G	11 (22.9%)	29 (93.0%)	
MARCO	rs1318645	CC	14 (58.3%)	4 (17.4%)	0.002^#^
		CG	9 (37.5%)	9 (39.1%)	
		GG	1 (4.2%)	10 (43.5%)	

* *Allelic frequencies;*

# *genotype frequencies*.

## Discussion

Respiratory viruses are one of the causes of early life wheezing that may contribute to the development of childhood asthma. Several epidemiological studies reported that respiratory virus-associated wheezing during early life, particularly during infancy, contributes to the development of asthma during childhood (9–12).

Growing data have associated specific SNPs in several immune-related genes with altered susceptibility to infectious diseases, but despite their potential role, there are few data about SNPs associated with RSV and other respiratory infections; a systematic literature review has been performed by Drysdale et al. (13).

SNPs in genes coding for interleukin (IL)-8, IL-19, IL-20, IL-13 mannose-binding lectin, interferon-gamma, and a regulated on activation, normal T cell expressed and secreted polymorphism have been associated with subsequent wheeze after RSV lower respiratory tract infection in term-born infants (14). Utilizing the inpatient database of severe RSV infection or without, we compared MARCO rs1318645 [GenBank access number NT_005403.18^2^] polymorphism and risk of recurrent wheezing or recurrent airway infections during childhood. Our finding allowed us to identify a biologically plausible bronchiolitis susceptibility gene candidate: MARCO. Unexpectedly, we identified the wild-type C allele frequency as an increased risk of recurrent infection and wheezing incidence in an infant. We did not show genotypic or allelic different susceptibility in bronchiolitis RSV positive vs. RSV negative, and, in contrast to what High et al. (15) reported, this research did not find evidence supporting a causative role of C/C genotype o G allele for RSV disease severity. In fact, they determined that G allele and G/G genotypes were more likely to have severe RSV disease than children who were heterozygous or wild type for the C allele, showing a missense loss of function polymorphism in the human MARCO homolog associated with severe RSV disease in two populations of infected infants (15). Demonstrating a correlation between the wild-type C allele and increased susceptibility to bronchiolitis, we can speculate an important role of MARCO in the pathogenesis of airway infection, but our results about RSV disease severity differ from those of High et al. suggesting that the C allele increases the risk of bronchiolitis but in a mild presentation, whereas G allele is associated with severe clinical form of the disease. The precise role of MARCO is still not clear in our study, as well as the utility of more studies for understanding human RSV disease.

Although RSV infection is very common, a minority of infected children develop wheezing during childhood. The genetic factors that increase the risk of wheezing have not been clarified yet.

Respiratory viruses replicate in cells, damaging airway epithelium that is a chemical, physical, and immunological barrier. They cause loss of cilia, decreasing mucociliary clearance and promoting the sensibilization to allergens and environmental irritants. Causing sustained airway inflammation, recurrent infections of the respiratory tract play a major role in the pathogenesis of wheezing (16), and one wheezing episode or more during the first years of life are confirmed risk factors for the development of asthma (2, 17, 18).

Also, the atopic clinical history and the early-life wheezing episodes synergistically contribute to asthma pathogenesis (19, 20).

We hypothesize the immune system impairment on the epithelial barrier, in which MARCO is involved, predisposing to respiratory tract infections and recurrent wheezing in the first years of life. For this reason, we established a follow-up of children affected by bronchiolitis to study wheezing development as a risk of future asthma. As shown in our results, we identified the wild-type C allele frequency and C/C genotypes as increased risk of recurrent infections and wheezing 5 years after the bronchiolitis episode rather than the G allele. This confirms that, unexpectedly, the wild-type allele in SNP rs1318645 is correlated with an increased susceptibility to respiratory sequelae of bronchiolitis. We also observed a trend in our results that describe a major susceptibility to recurrent wheezing in children with previous RSV + bronchiolitis. This is confirmed by literature from years (21), although the link between RSV infection and the development of wheezing is still partially unclear. At the basis of this phenomenon, it would seem to be involved not only in the remodeling of the respiratory tract after the inflammatory process. For this reason, researchers are focusing on SNPs, speculating about their genetic predisposition to atopy, wheezing, and asthma. For example, SNPs s8076131, rs12603332, and rs3744246 of gene ORMDL3, which has been reported to be involved in the lung epithelial defense pathway, have been associated with the development of asthma such as GSDMB SNPs rs7216389 (22, 23). Also, the SNPs rs1889570 (C/T), IL-17A rs4711998 (A/G), and IL-17A rs3819024 (A/G) of THE IL17F gene have recently emerged as associated with more post- bronchiolitis asthma development (24). ILs are also associated with allergic sensibilization, such as polymorphisms of the genes that code for IL-4 and IL-13 (25–27), such as a correlation between toll-like receptor 9 rs187084 gene polymorphism and wheezing has been described (28). From our study, apparently, the genetic predisposition seems to affect the airway infection susceptibility and the recurrent wheezing more than the previous RSV infection. As we can see, candidate gene SNPs are the vanguard for the future study of genetic contributions to complex, multifactorial diseases, specifically examining common variants and eventually making it possible to identify determinants of disease.

We admit that there are some limitations to our study. First, our research is focused on a single SNP (MARCO rs1318645 polymorphism), although nowadays, it is possible to investigate more polymorphisms, such as IL-4 rs2243250, ADRB2 rs1042713, and FCER1B rs569108 and IL-13 rs20541 (29, 30).

Besides, our methods of data collection do not include a respiratory function test, such as spirometry, to define the grade of bronchoconstriction in patients with wheezing.

A small number of subjects have been enrolled, and for this reason, it is necessary to explore the effects of other variables that might result in misleading interpretations of causality.

Nevertheless, our work remains relevant, as our findings contribute to the emerging global picture of recurrent wheezing and childhood-onset asthma and because it outlines the roots for future, more integrated studies.

In summary, we describe an association between MARCO rs1318645 polymorphisms and susceptibility to recurrent airway infection and recurrent wheezing in children, but mechanisms through which MARCO modulates the inflammatory response to RSV and the disease severity are not completely understood. As innate immunity receptors seem to play a critical role in host defense, as well as allergy, nutritional factors, and asthma, the genetic risk factors involved in susceptibility to RSV infections are needed to eventually prevent RSV disease and associated sequelae in children.

Future studies of genetic associations should consider the different wheezing phenotypes in infancy. In addition, stratified genetic analyses for subjects with atopy can be useful for elucidating the background mechanisms of recurrent wheezing.

## Data Availability Statement

The original contributions presented in the study are included in the article/supplementary material, further inquiries can be directed to the corresponding author/s.

## Ethics Statement

Ethical approval was not provided for this study on human participants because the work described was carried out in accordance with The Code of Ethics of the World Medical Association (Declaration of Helsinki) for experiments involving humans, http://www.wma.net/en/20activities/10ethics/10helsinki/index.html. Written informed consent to participate in this study was provided by the participants' legal guardian/next of kin.

## Author Contributions

The guarantors of the study are FS and MB, from conception and design to conduct of the study and acquisition of data, data analysis, and interpretation of data. FS, FP, and MB have written the first draft of the manuscript. FP has performed follow-up, tables, and searched references. VD, CC, CA, PM, and IG have performed genetic investigations. All co-authors have provided important intellectual input and contributed considerably to the analyses and interpretation of the data. All authors guarantee that the accuracy and integrity of any part of the work have been appropriately investigated and resolved, and all have read, edited, and approved the final version of the manuscript. The corresponding author had full access to the data and had final responsibility for the decision to submit for publication. No honorarium or other forms of payment was given to any of the authors to produce this manuscript.

## Conflict of Interest

The authors declare that the research was conducted in the absence of any commercial or financial relationships that could be construed as a potential conflict of interest.

## Publisher's Note

All claims expressed in this article are solely those of the authors and do not necessarily represent those of their affiliated organizations, or those of the publisher, the editors and the reviewers. Any product that may be evaluated in this article, or claim that may be made by its manufacturer, is not guaranteed or endorsed by the publisher.

## Footnotes

^1^Available online at: https://www.wma.net/policies-post/wma-declaration-of-helsinki-ethical-principles-for-medical-research-involving-human-subjects/

^2^Available online at: https://ftp.ncbi.nlm.nih.gov/genbank/
